# Supplementary material for: Are carbon emissions trading and green financial instruments synergistic? -Comprehensive quantitative research based on content analysis
Source: PLoS One. 2024 Mar 7;19(3):e0298601. doi: 10.1371/journal.pone.0298601 (PMC10919601; doi:10.1371/journal.pone.0298601)
Supplement: S1 Data — (ZIP) [file pone.0298601.s001.zip › Data/地级市Z_F工作报告.zip!/╣π╢1⁄2/╗▌╓▌╩╨/└┤╫╘╣1⁄2╓┌║┼í╛┬φ┐╦╩2╛▌═°í┐_2013.docx]

各位代表：

　　现在，我代表市人民政府向大会报告政府工作，请予审议，并请政协各位委员和其他列席人员提出意见。

　　2012年工作回顾

　　2012年是惠州的发展之年、改革之年、稳定之年、丰收之年。在省委、省政府和市委的正确领导下，在市人大及其常委会和市政协的监督支持下，市政府紧紧围绕主题主线，以转型升级为路径，以改革开放为动力，以改善民生为目的，全力推进科学发展惠民之州建设，经济发展逆势上扬，重点改革不断深化，民生质量明显提升，市十一届人大一次会议确定的目标任务圆满完成。

　　一、有效提升经济发展质量，综合实力再上新台阶

　　深入实施扩内需、稳外需和帮扶企业发展等一系列措施，经济发展态势良好。

　　发展速度和质量同步提升。实现生产总值2368亿元，比上年（下同）增长12.6%，增幅居珠三角首位；人均生产总值50884元，增长11.7%。公共财政预算收入200.88亿元，增长23.37%，增幅居珠三角首位。城镇居民人均可支配收入29965元，农民人均纯收入12415元，分别增长12.6%和13.5%。居民消费价格指数上涨2.8%，在4%的控制目标内。

　　投资、消费、出口协调拉动。引进合同资金1092亿元，成功举办哈尔滨、长沙和巴西圣保罗展销会，签约金额362亿元。完成固定资产投资1208.7亿元，增长18%，总量跃居全省第四。社会消费品零售总额754.2亿元，增长15.5%，增幅居珠三角第二。外贸出口292亿美元，增长26.3%，增幅居全省首位。

　　实体经济稳步发展。出台28条扶持措施，开展“企业服务月”活动，为企业减免税费3.5亿元，安排扶持资金3.6亿元。工业企业经济效益综合指数提高20.5个百分点。47家规模以上企业增资扩产。新增私营企业6896家、个体工商户3.2万户。农业产业化加快推进，全市各级农业龙头企业发展到230家，带动农户23万多户，户均增收4500元左右。

　　二、大力推进产业转型升级，产业结构呈现新格局

　　充分发挥龙头项目带动作用，深入实施自主创新战略，产业竞争力持续增强。

　　产业结构优化升级。服务业比重由36%提高到36.5%，先进制造业增加值占规模以上工业比重由65.6%提高到66.7%。石化、电子产业向集聚化高级化发展。中海油丙烯酸及酯、巴斯夫丁苯胶乳等项目投产。石化产业实现增加值311.5亿元，增长16.2%。仲恺高新区获批国家LED产业基地、新型工业化产业示范基地。成功举办“云博会”。电子信息产业实现增加值440.4亿元，增长27.8%。惠州港年吞吐量突破7000万吨。实现旅游总收入184.2亿元，增长14.3%。金融机构本外币存、贷款余额分别比年初增长12.3%、20.6%，存贷比达64.3%，获省“金融稳定奖”。

　　科技创新能力增强。新增国家级创新平台1个 [1]，新组建省部产学研联盟3个，引进科技创新团队5个。预计研发经费支出占GDP的2%。荣获省科技进步奖7项，申请专利9894件。新增驰名商标4件、高新技术企业28家、省级创新型企业8家，高新技术产品产值占规模以上工业的48.6%，占比居全省第二。

　　节能减排成效明显。关停重金属污染企业113个。单位GDP水耗下降10.6%。超额完成省下达节能降耗任务，省节能考核获评优秀。主要污染物排放控制在省下达目标之内。仲恺高新科技产业园获认定“省市共建循环经济产业基地”。

　　三、全面深化重点领域改革，体制机制焕发新活力

　　坚持市场化的改革方向，系统推进配套改革，政府公共服务总体满意度居全省第一 [2]。

　　经济体制改革取得重大突破。实施商事登记制度改革，审批提速85%，工商登记增长65%。成立市公共资源交易中心，28类公共资源实现集中交易，为财政增收节支5.6亿元。承担全省“两建”[3]试点，率先建成“一库两网三平台”[4]，成立市信用中心。组建市交通投资集团、水务集团，新增4家创业投资机构，启动投资项目审批制度改革。

　　行政体制改革深入推进。完成第五轮行政审批制度改革，市级行政许可由211项减少到70项，全部实行 “零收费”。建成并开通市、县（区）网上办事大厅，市直966项服务事项实现网上办理，占比94.3%；县（区）3093项服务事项实现网上办理，占比53.2%。实办业务200多万宗。

　　社会体制改革有序开展。登记注册社会组织1698家。实行政府向社会组织购买服务，首批购买服务262项。建成全省首家社区服务网络中心，“特色之家”入选全省社会管理十大创新项目。2万多名异地务工人员通过积分制入户城镇。

　　基本公共服务均等化综合改革成效初显。在全省率先开展综合改革试点，市级208项、县区1189项目标任务全面完成，初步建立政府主导的多元投入机制、由民作主的决策机制、底线均等的保障机制和均衡发展的资源配置机制。全市财政基本公共服务支出95.8亿元，增长29.5%，增幅高出同期财政预算支出9个百分点。

　　四、着力统筹城乡区域协调发展，“三宜”惠州展现新面貌

　　深入实施《珠三角规划纲要》，扎实推进城乡区域一体化，“宜居宜业宜游”的特色更加突显。

　　城市功能进一步完善。建成一批城市道路、连接路和立交设施 [5]。启动创建国家森林城市，建设园林风景项目36个。建成望江公园、金山湖公园（一期）和龙丰公园。新增绿道152.8公里。人均公园绿地面积15.8平方米。新增公交线路25条，建成便民自行车服务站点100个，投放自行车1万辆。城市管理实现网格化 [6]。

　　城乡区域协调发展。莞惠城轨、厦深铁路加快建设。广河高速建成通车，提前四年实现“县县通高速”。完成县道改造50公里、农村公路硬底化800公里。新增5个省级宜居示范城镇、12个省级宜居示范村庄，建成鹿颈村岭南新民居示范点。实现“村村通光纤”。完成深莞惠区域协调发展总体规划，新开通3条跨市公交线路。龙门、惠东两个产业转移园新引进项目14宗，总投资25.8亿元，省产业转移考核获评优秀。实施《珠三角规划纲要》2011年度考核获珠三角第一。

　　生态环境保护成效明显。圆满完成省人大确定的“两河”[7]流域污染整治任务，建成金山河整治主体工程。新建城镇污水处理设施21座、垃圾无害化处理场1座，县县建成垃圾处理场。新上项目环保否决率11.9%。新增省级生态乡镇7个。森林覆盖率60.87%。通过国家环保模范城市复核，省环保责任考核连续五年获评优秀。

　　五、切实保障和改善民生，社会建设得到新提升

　　全市公共财政民生支出183.33亿元，增长26.2%，占财政预算支出比重由63.9%提高到66.9%，十件民生实事基本完成，群众幸福感总体评价居珠三角首位 [8]。

　　公共文化教育更加完善。启动申报国家历史文化名城，完成广播电视“村村通”工程，免费开放公共文化场馆和市区公园景点，成功举办一批高水平文艺演出和体育赛事。实现“镇镇皆教育强镇”、“县县有示范高中”、乡镇中心幼儿园全部规范化。市东江高级中学建成。中职新城完成年度建设任务 [9]。高考新增本科上线人数占全省的1/5。57.6%的异地务工人员随迁子女接受公办义务教育。

　　医疗保障大幅提升。建成市中医医院新院、中大惠亚医院，完成市中心医院改扩建一期工程、第三人民医院综合大楼及329间基层医疗卫生机构标准化建设。第四人民医院成为全省首家平价二级医院，115家医疗机构开展“平价药包”服务。巩固完善基本药物制度和基层综改运行新机制，实施镇村卫生服务一体化管理。基本实现全民医保，人均医保补助从210元提高到252元；居民医保政策内平均报销比例由70%提高到80%，最高支付限额由12万元提高到30万元；职工医保政策内报销比例由90%提高到95%。

　　社会保障不断增强。新增城镇就业岗位7.3万个，农村劳动力转移就业1.54万人，城镇登记失业率控制在2.35%。城乡居民养老保险全覆盖。老龄津贴提高一倍，新发放老年归侨补贴和困难残疾人补贴 [10]。城乡低保由城镇最低每月270元、农村最低210元统一提高到385元。率先实施低收入群体燃气价格补贴。建成保障性住房5400套，完成农村困难户住房改造4372户。发放公积金贷款22.4亿元，增长95%。74个省级贫困村和12270户贫困户实现脱贫。

　　平安惠州建设扎实推进。深入开展法治城市建设，荣获全省首批法治城市创建先进单位。“三打”取得积极成效 [11]，绩效考核总分排名全省第四，获评优秀档次。全警上路大巡防全面铺开，综治信访维稳网络更加完善，安全生产、食品药品安全监管进一步加强，社会和谐稳定，群众安全感不断提高。

　　社会各项事业加快发展。通过全国公共文明指数测评，获评全国“未成年人思想道德建设工作先进地区”。成功创建全国“计划生育优质服务先进市”。蝉联“全国双拥模范城”四连冠。与巴西圣保罗、智利维尼亚德尔玛市开展友好交往。国防教育、人民防空、民兵预备役工作迈出新步伐，妇女儿童、老年人、残疾人事业取得新发展。统计、审计、外事、侨务、台务、民族、宗教、方志、档案、移民、仲裁、气象、三防、消防、应急、防震、打私等工作取得新进步。

　　在推动经济社会快速发展的同时，政府自身建设不断加强。认真执行市人大及其常委会决议并报告工作，自觉接受人民政协民主监督。办理人大代表议案13件、建议134件，政协提案139件、委员意见建议126件，办理网民来信3.5万件，办复率均为100%。广泛开展“党政领导信箱”、“行风热线”、“惠民在线”等活动，开通政府官方微博，建成覆盖县区和部门的政务微博群。第三次获评“广东网络问政年度城市”。依法行政工作有效开展，党风廉政建设深入推进，政府效率和服务水平不断提升。

　　各位代表！过去一年的成绩来之不易。这是省委、省政府和市委正确领导的结果，是全市人民共同奋斗的结果，是海内外各界朋友关心支持的结果。在此，我代表惠州市人民政府，向在各个岗位上辛勤劳动的全市广大工人、农民、知识分子、干部职工，向给予政府工作大力支持的人大代表和政协委员，向驻惠部队、武警官兵、人民警察和各民主党派、人民团体、社会各界人士致以崇高敬意！向所有关心支持我市改革发展的港澳同胞、台湾同胞、海外侨胞和国际友人表示衷心感谢！

　　我们也清醒地看到，我市经济社会发展中仍然存在不少矛盾和问题，主要有：人均GDP、城乡居民收入落后于珠三角平均水平，自主创新能力有待增强，产业结构重型化给节能减排带来严峻考验，部分企业生产经营困难，城乡区域基本公共服务均等化水平有待提高，社会管理面临很多新情况和新问题。对此，我们要高度重视，切实采取有效措施认真加以解决。

　　2013年工作安排

　　2013年是贯彻落实党的十八大精神的开局之年，也是我市重点项目推进年、惠民实事建设年、转型升级提速年。综观当前宏观经济形势，虽然经济企稳回升的基础尚不牢固，经济下行压力较大，但保持稳中求进的态势仍然具有许多积极因素和有利条件，我们仍然处于可以大有作为的重要战略机遇期。党的十八大胜利召开，描绘了全面建成小康社会、夺取中国特色社会主义新胜利的宏伟蓝图；习近平总书记视察广东，提出了广东发展“三个定位、两个率先”的总目标，为广东发展指明了前进的方向；胡春华书记莅临惠州考察调研，寄予我市“尽快进入珠三角第二梯队”的殷切希望，明确了我市当前和今后一段时期经济社会发展的总目标、总任务，极大鼓舞了全市干部群众。我市一大批重大项目建成投产或动工建设，为惠州在新的起点上加快发展、转型升级，奠定了坚实基础，注入了强劲动力。与此同时，全省各地正在掀起新一轮加快发展的热潮，百舸争流，不进则退，慢进也是退。面对新形势、新目标，我们必须进一步增强推进科学发展的自觉性和坚定性，进一步增强建设惠民之州的责任感和紧迫感，坚定不移加快发展，坚定不移加快转型，以更加高昂的斗志、更加务实的作风、更加有力的举措、更加扎实的工作，全面开创我市科学发展、加快发展的新局面。

　　政府工作的总体要求是：全面贯彻落实党的十八大、习近平总书记视察广东重要讲话、胡春华书记调研惠州重要讲话和市第十次党代会第二次会议精神，紧紧围绕“尽快进入珠三角第二梯队”的总目标，突出主题主线，坚持“稳中求进、好中求快、率先跨越”，全力推进“重大平台、重大产业、重点企业”建设，深化改革开放，加快转型升级，促进生态文明，强化民生保障，争先进位，率先跨越，努力实现更快速度的发展、更有质量的转型、更高水平的惠民，为2017年率先实现“两个翻番”[12]、全面建成小康社会打下更加坚实的基础。

　　经济社会发展的主要预期目标是：生产总值增长11%；人均生产总值增长10%；固定资产投资增长16%；社会消费品零售总额增长15%；外贸出口增长10%；地方公共财政预算收入增长13%；城镇居民人均可支配收入增长11%；农民人均纯收入增长12%。居民消费价格指数控制在103.5%以内；服务业增加值占生产总值的36.5%左右；城镇登记失业率控制在2.7%以内；户籍人口自然增长率控制在9.2‰以内；全社会研发经费支出占生产总值比重2.0%以上；节能减排完成省下达任务。

　　围绕上述目标，必须着力推动“八个率先跨越”：

　　一、在壮大经济实力上率先跨越

　　坚持扩消费、促投资、拓外需并举，做强企业，做大总量，提升质量，促进经济持续健康发展。

　　强化投资拉动作用。开展好“重点项目建设年”活动，发挥重点项目带动作用，安排重点项目170项，年度计划投资414.8亿元。抓好交通、能源、环保、水务、市政等重点项目建设，实现基础设施投资稳步增长。积极拓宽民间投资，推动企业增资扩产，鼓励技改和创新投入。加强招商选资，力争引进一批带动能力强的优质项目。切实提高已签约项目履约率。

　　有效扩大消费需求。稳步增加城乡居民收入，提升消费能力。加快落实国家鼓励消费政策，推进特色商业街区、专业市场建设改造，完善农村商业网点，扩大城乡消费。拓展社区服务、文化休闲、旅游度假等热点消费，鼓励发展网购，推广信贷、租赁等新型消费模式。办好惠州产品呼和浩特、俄罗斯展销会，建成电子商务服务中心，提高惠货市场占有率。

　　保持外贸快速增长。全面落实稳出口各项政策，大力帮扶重点出口企业。深度挖掘传统市场潜力，积极开拓东盟、俄罗斯、南美、中东等新兴市场。积极扶持高新技术、自主品牌产品和一般贸易出口，大力发展服务贸易，鼓励建设境外营销网络和对外投资带动出口。扩大先进技术设备、关键零部件和资源型产品进口。加快推进加工贸易转型升级。深化与港澳台的经贸合作交流。推进口岸建设与开放，提高通关效率。

　　强化金融支撑作用。积极发展现代金融业，大力引进各类功能性金融机构，完成组建惠东、博罗农商行，启动组建龙门农商行。加强金融、科技、产业三融合，充分发挥金融推动科技创新、产业升级的促进作用。整合产业、科技扶持资金，加快发展创业投资，组建2家创投机构。积极推动企业上市和股权融资，完成3家企业上市。加强政银企合作，更好发挥交通投资集团、水务集团等融资平台的作用，加大优势产业、重点项目的融资力度，强化金融对地方经济发展的支撑。

　　实施企业成长计划。发展壮大龙头企业和企业集团，培育年主营业务千亿元企业1家，新增超百亿元企业1家。重点扶持100家创新型、成长型企业加快发展。完善中小微企业综合服务平台 [13]和信贷支持政策，支持中小微企业与大企业合作配套，提升企业整体竞争力。深入实施民营企业自主创新能力和企业家素质提升工程，推动民营企业转型升级、发展壮大。

　　加强经济运行调节。实施扶持实体经济发展的新“惠28”条措施。完善经济运行监测分析例会制度，提高生产要素的调节和保障能力。加强财税金融监测预警，提高财税管理科学化、精细化水平。落实重要商品的储备管理和产销运衔接，稳定物价总水平。引导房地产市场健康发展。

　　二、在构建现代产业体系上率先跨越

　　实施“产业培育计划”，大力发展重大产业，加快构建现代产业体系，进一步提升产业竞争力。

　　做大做强“电、汽、化”产业。开工建设中海油炼化二期项目，大力引进石化中下游项目特别是精细化工项目，推进石化产业链延伸发展。加快TCL液晶、移动互联网、“慧云”生态创意等产业园和北斗装备制造应用产业基地建设，推动电子信息产业向高端化发展。规划建设博罗、惠阳、大亚湾汽车产业园区，争取引进有实力的企业，做大汽车产业。深入实施“两化融合牵手工程”[14]，推动传统产业转型升级。

　　发展壮大战略性新兴产业。依托省市共建战略性新兴产业基地，大力发展LED、物联网和云计算应用等创新型产业集群，加快培育发展新材料、新能源、生物制药等新兴产业。推进绿色照明示范城市、新能源汽车推广应用示范城市建设。巩固首届“云博会”成果，办好第二届“云博会”。

　　优先发展现代服务业。着力推进现代服务业集聚区建设。加快荃湾港区国际集装箱码头二号泊位、煤炭码头一期等项目建设，推进铁水联运，扩大货源，打造亿吨大港。加快惠州机场和“空港”经济区建设。推进罗浮山、西湖创“5A”。培育发展健康卫生、智慧生活、绿色环保、文化创意等幸福导向型产业。

　　加快发展现代农业。建设高标准农田25万亩，加快现代农业综合示范区建设。以十二大特色农产品 [15]为重点，积极发展品牌农业、设施农业、观光农业和外向型农业。完善农业科技、信息服务体系，推进精深加工和冷冻保鲜等技术应用，扶持一批农业龙头企业和农民专业合作社，促进农业增效、农民增收。

　　三、在创新驱动发展上率先跨越

　　积极构建多元化创新体系，加快科技成果应用开发和产业化，打造珠三角科技成果产业化基地。

　　健全区域创新体系。加快构建以企业为主体、产学研结合的区域创新体系。进一步落实企业研发税收优惠政策，鼓励和支持企业加大研发投入。加快大亚湾石油化工研究院、仲恺电子信息研究院等公共创新平台建设，新增技术创新平台15家。从今年起连续三年每年投入1000万元，支持专业镇建设公共服务平台。实施“双高人才计划”，大力培养和引进科技领军人才和高层次专业人才。

　　加快科技成果产业化。优化科技财政投入，鼓励应用研究和成果产业化。加快科技企业孵化器群建设，发展科技综合服务，促进创新创业。发挥产业集聚优势，积极承接先进地区的技术与产业溢出。推广合同能源管理 [16]、重大技术设备融资租赁 [17]等新型商业模式，支持新产品开发应用。加快发展高新技术产业，争取新认定高新技术企业18家。

　　实施知识产权、技术标准和名牌带动战略。创建全国知识产权示范城市，力争专利申请量和授权量分别增长20%以上。支持优势产业建立知识产权联盟，推进技术专利化、专利标准化、标准产业化。深入推进品牌带动战略，力争新增驰名商标4件。

　　四、在体制机制创新上率先跨越

　　坚持全局和局部相配套、治本和治标相结合、渐进和突破相促进，以更大勇气和智慧推动重点领域改革。

　　深化经济体制改革。系统推进投资项目审批制度改革，实现审批提速80%以上 [18]。继续推进商事登记制度改革，实现市、县（区）有效衔接。完善公共资源交易体制，完成所有中心镇公共资源交易所建设。加强社会信用体系和市场监管体系应用系统的建设推广，优化营商环境。进一步完善市与区的财政体制，探索建立专项资金竞争性分配制度。做好营业税改增值税试点。深化国企改革，促进国有资产保值增值。推进资源环境价格改革，探索水价“全成本”定价和区域供水同网同价。

　　深化行政体制改革。继续推进行政审批制度改革，行政审批事项压减40%以上，加快行政审批标准化建设。完善网上办事大厅，确保所有行政审批及公共服务事项都可实现网上办理。深化简政富县强镇事权改革。

　　深化社会管理创新。推进社会管理体制改革，加快建设民生社会、平安社会、公平社会、活力社会、法治社会。建成社会组织“孵化器”，扶持120家以上社会组织发展。完善政府向社会组织购买服务制度。强化工青妇等群团组织的枢纽功能，开展社区社会工作服务试点，全面推进社区“六个一”工程 [19]。实施“千名社工人才培养计划”，发展社工队伍，引导城市社区服务资源向农村延伸。创新和完善出租屋、流动人口服务管理。

　　深化农村综合改革。以惠东和龙门为试点，推进主体功能区建设，实行配套的财政、产业、土地、环境政策和考核制度，探索山区发展新路子。稳步实施农村集体资产产权制度改革，推进资产确权登记，鼓励对集体资产统一管理和交易。稳步推进农村集体土地管理制度改革，探索建立城乡统一的建设用地市场。推行村（居）自治事务与经济事务相分离。深化华侨农场改革。

　　五、在城乡区域协调发展上率先跨越

　　推进城乡规划和基础设施一体化，促进县区联动发展，积极参与深莞惠经济圈建设，进一步缩小城乡区域发展差距。

　　着力提升城镇化质量。加快中心城区扩容提质，优化“一城三组团”功能布局，增强辐射带动力。提升惠东、博罗县城建设水平，逐步与市区融合发展。推进金龙大道沿线、龙门县城建设和生态旅游开发，打造北部生态发展组团。加快中心镇、专业镇和名镇名村建设，推进基础设施、公共设施与周边镇村共建共享。有序推进农业转移人口市民化。建设流动商户临时疏导区。

　　大力推进信息化建设。加快信息技术的拓展和应用，打造信息化高地，建设智慧城市 [20]。拓展惠民卡功能和使用范围，建成“读者云图书馆”和电子政务云数据中心一期工程，建设住房公积金运营与监管系统、数字城管系统和社会管理综合信息系统，提高城市管理信息化水平。

　　统筹城乡基础设施建设。启动轨道交通规划。加快莞惠城轨、厦深铁路和7条在建高速公路 [21]建设，完成国道205线和省道357线惠州段以及三环路等路面改造。抓紧四环路南段、金恺大道、惠新大道等城市主干道建设。启动云山东江大桥、第三东江大桥、新开河北桥建设。推进惠州大堤南堤堤路贯通、潼湖围达标加固和观洞水库应急供水扩容等工程。加快灌区和小农水改造。抓好祯州、现代、崇文、尾塘等输变电工程。

　　加快环大亚湾新区建设。编制完成环大亚湾新区开发建设总体规划、空间发展规划、干线路网规划，修编全市海洋功能区划，启动编制海岸保护利用规划。整合沿海港口、岸线和产业资源，优化空间布局，促进产业集聚、配套发展，打造新的增长极。加快区域性基础设施和公共服务设施建设，促进区域融合发展。

　　推进深莞惠经济圈建设。全面落实合作协议和重点事项。推进深惠城轨前期研究，加快龙江东江大桥、县道225线惠阳秋湖路口至深圳坑梓段改造等项目建设。争取开通大亚湾至大鹏、新圩至龙岗等跨市公交。完善环保联防联治机制，深入推进“两河”污染整治。完善龙门、惠东产业转移园基础设施、产业布局，引进更多优质项目。推进坪惠合作区 [22]规划建设。

　　六、在生态文明建设上率先跨越

　　坚持资源节约集约利用，加强生态建设和环境保护，推进绿色、循环、低碳发展，建设美丽惠州。

　　促进资源节约集约利用。启动市能源管理中心建设，推广应用节能减排技术，引导企业节约用电用水。鼓励企业开展清洁生产，狠抓水泥、石化等重点行业节能降耗。合理控制土地开发强度，提高用地效能。大力推进“三旧”改造，积极开展低丘缓坡地开发利用试点。加强水源地保护和用水总量控制。

　　深入推进治污减排。完成金山河综合整治，推进青年河等重点河涌治理。新建21座污水处理设施，确保已建污水处理设施正常运行。实行主要污染物排放总量前置审批。推进重污染企业淘汰，全面淘汰中小燃煤锅炉，完成水泥生产线降氮脱硝改造。加强养殖业和机动车减排。统筹城乡生活垃圾无害化处理。

　　提高生态建设水平。深入开展生态县、生态镇、生态村创建活动，加快规划建设潼湖湿地公园，抓好海洋生态园建设和考洲洋生态修复，建设海洋生态文明示范区，推动国家生态市创建工作。大力推进生态景观林带、森林碳汇和森林进城围城工程建设，实施中心城区和县城中心区造林绿化工程，完成8个森林公园、100个森林家园和7个森林绣花工程建设，确保建成区绿地率、绿化覆盖率在36%、40%以上。实施村镇“绿色家园工程”，建成10个“千村家园绿化示范村”。力争成功创建国家森林城市，在生态建设上争当珠三角“第一梯队”。

　　七、在文化建设发展上率先跨越

　　以提升全民素质为核心，深入推进文明创建，大力发展文化事业，积极培育文化产业，全面提升城市文化软实力。

　　深化文明城市建设。围绕夺取全国文明城市三连冠，广泛开展 “共筑中国梦、当好建设者”宣传教育活动，大力弘扬新时期广东精神、惠州精神，加强社会公德、职业道德、家庭美德、个人品德和未成年人思想道德建设。倡导社会志愿服务和爱心互助，推进“好人之城”、“道德讲堂”建设，提升市民整体素质和城市文明程度。加大城市综合整治力度，确保顺利通过全国公共文明指数测评。

　　申报国家历史文化名城。保护和修缮一批古街区、古村落、古建筑。科学规划、保护和整治水东街、金带街等历史文化街区，加强对银岗窑址 [23]、罗浮山葛洪炼丹遗迹、宾兴馆等历史遗存的保护修缮，启动重修东坡祠。加强古迹保护，研究甄别和形成《惠州市古籍联合目录》。积极推进非物质文化遗产的保护利用。

　　优化公共文化服务。重点推进县（区）、乡镇（街道）、行政村（社区）公共文化设施建设和达标升级。完善公共文化场馆免费开放机制，推进广播电视“户户通”。建设市数字图书馆，推行图书借阅“一卡通”[24]，广泛开展送戏下乡、送书下乡、公益电影放映等文化惠民活动。开展“文化惠民卡”试点，保障困难群体的基本文化权益。

　　培育发展文化产业。充分挖掘特色文化资源，推动文化与商贸、旅游、科技结合，重点推进龙门农民画产业园、读者（惠州）数字出版基地、雅图光电和数字视频产业基地、罗浮山文化产业孵化基地、艾米动漫基地等一批文化产业项目建设。实施“原创动漫扶持计划”，提高原创能力和文化创意水平。

　　八、在提高民生质量上率先跨越

　　巩固和扩大基本公共服务均等化综合改革试点成果，力争提前两年实现国家基本公共服务体系“十二五”规划目标。

　　完善就业和社会保障。全面落实就业扶持政策，促进城乡居民更加充分就业。继续抓好社会保险扩面提标，完善社会保障卡应用功能。推进市社会福利服务中心建设，推广社区居家养老服务。落实低收入群体物价补贴双联动机制，保障困难群众基本生活。推进新一轮扶贫开发工作。

　　推进教育创强争先。巩固省教育强市创建成果，确保惠阳区和16个镇全部通过省教育强区、教育强镇复评。抓好创建省推进教育现代化先进市工作。加大农村教育投入，促进各级各类教育协调发展，争取县（区）全部创建为国家义务教育发展基本均衡县（区）。申办1所高职院校，建成市技师学院二期工程。推进规范化幼儿园和民办义务教育规范化学校建设。实施“强师”工程，培育一批教学名师和优秀教学团队。

　　提升医疗卫生服务水平。启动健康新城一期工程建设。推进市第一和第三人民医院等综合性医院创“三甲”工作，确保市中心医院、市中医医院“三甲”复审、县（区）中医医院“二甲”复审达标。深化县级公立医院综合改革试点工作，深入推进镇村卫生服务一体化管理，扩大平价医疗服务。扶持民营医疗机构发展。迁建市人口计生服务中心。打造卫生信息平台，建设“智慧医疗”和“全民低成本健康海云工程”[25]，实现健康管理全民覆盖。

　　深化平安惠州、和谐惠州建设。构建“两圈五网”[26]立体化治安防控体系，维护社会治安稳定。严厉打击涉毒违法犯罪，确保禁毒严打专项行动取得明显实效。深入开展“六五”普法。完善社会矛盾纠纷大调解机制，层层落实信访工作责任，依法妥善处置突发性、群体性事件。落实安全生产责任管理，加强重点领域专项整治，严防重特大事故发生。加强食品药品市场监管。

　　统筹发展各项事业。广泛开展群众体育运动，筹办第四届市运会、第二届国际龙舟邀请赛、第十三届世界象棋锦标赛、第五届省少数民族传统体育运动会等体育赛事。全面推进“惠一家”优生健康工程 [27]，推广生育关怀基金。创建防震减灾示范市。建设市档案馆二期工程和各县档案馆新馆。组织实施第三次全国经济普查。继续做好双拥共建、人民防空、外事侨务、民族宗教、审计、三防、应急、台务、仲裁、方志、科普、打私、老龄、残疾人等工作。

　　各位代表！保障和改善民生，建设惠民之州，是我们始终如一的追求。从今年起，市本级新增财力75%以上、县级新增财力60%以上投入民生。今年深入开展“惠民实事建设年”活动，我们将集中力量办好十件民生实事：

　　一是创造更多就业机会。新增城镇就业岗位6万个，帮助3000名就业困难人员再就业、1万名农村劳动力转移就业。

　　二是提高社会保障水平。建立并完善重特大疾病医疗救助专项制度，实施商业保险大病二次补偿；将儿童白血病、艾滋病机会性感染、慢性粒细胞白血病、脑梗死和甲状腺功能亢进性心脏病5种疾病纳入医保门诊特定病种；提高城乡低保标准；提高城乡居民基础养老金标准。

　　三是提供更好的受教育条件。在惠城、惠阳、大亚湾、仲恺4个区开展创建省推进教育现代化先进区工作；新增和改扩建一批公办中小学校 [28]，新增小学学位8434个、初高中学位2840个；实施城镇贫困家庭和农村义务教育学生“营养午餐计划”，对符合条件的在校学生给予午餐补助。

　　四是减轻看病就医压力。建设市中心人民医院改扩建二期工程；启动市第一人民医院扩建工程；在8间二级以上公立综合医院设立平价诊室，新增8间政府办医疗卫生机构使用平价中医适宜技术治疗包，新建30家以上平价药店；将城乡居民电子健康档案建档率提高到85%，增强档案使用功能。

　　五是缓解群众住房困难。筹建2165套公共租赁住房，完成4348户农村住房困难户住房改造和剩余的428户大中型水库移民住房改造，改造华侨农场危房1000户。

　　六是改善道路交通状况。完成800公里通自然村公路硬底化、50公里县道沙土路改造。打通市区10条市政连接路 [29]，新建5座人行过街设施 [30]，实施红花湖路、金榜路、梅湖大道等道路拓宽改造工程；新开通15条公交线路、新投放230辆公交车（其中惠城中心区环保公交车100辆）；在金山河镜河筛月、白鹭栖霞绿地配套建设地下公共停车库，金山河沿岸增设地面公共停车位，使市区新增一批停车位。

　　七是优化供水供气服务。建成日供水20万吨的潼湖水厂主体工程；开展大亚湾、惠阳、仲恺和惠城区供水一体化工作；新建16宗农村饮水安全工程，实现49个省级贫困村村村通自来水。完成惠城区、仲恺高新区3.5万户居民天然气转换。

　　八是提升宜居环境。建成8座污水处理厂 [31]并投入运行，完善2座污水处理厂配套管网 [32]。升级改造市垃圾焚烧发电厂和生活垃圾综合处理场；在惠城中心区逐步推行垃圾分类处理；完成镇街垃圾转运站、自然村垃圾收集点建设；新建生态景观林带192.4公里、碳汇林 [33]12.76万亩；城市添景增绿343.6万平方米。启动惠州市植物园建设。

　　九是提升群众安全感。深化“全警上路大巡防”，进一步提高路面见警率和查控率，实现市区重点区域全时空覆盖。新建300个、改造2610个高清视频监控点，提升视频监控系统实战效能；在市区治安复杂地段增设5个治安岗亭，日均投入18辆以上流动警务车，并常态化排查整治治安乱点。

　　十是完善基层便民服务。建设20个社区综合服务中心；新建60家平价商店；建成10个乡镇和60个自然村的体育健身设施；完成有线电视未通达的56795户广播电视“户户通”工程。

　　各位代表！惠州已经进入了科学发展、跨越发展的新时代。站在继往开来的新起点上，面对人民的新期盼，我们深感前景广阔、使命光荣、责任重大。全面加强政府自身建设，勇挑历史重任，势在必行。我们要全面落实中央关于改进工作作风、密切联系群众的八项规定，深入开展以为民务实清廉为主要内容的群众路线教育实践活动和“服务型机关建设年”活动。大兴实干之风，践行为民之举，力戒形式主义、官僚主义、享乐主义。大力推进政府职能转变，创新服务方式，优化政务服务，不断提升政府工作效率和服务水平。大力推进廉政建设，让权力在阳光下运行，力促干部清正、政府清廉、政治清明，坚持从严治政、依法行政，着力打造法治型、服务型、责任型、效能型、和谐型、廉洁型政府。

　　各位代表，时代赋予我们重任，人民寄予我们厚望。让我们紧密团结在以习近平同志为总书记的党中央周围，在中共惠州市委的坚强领导下，在人大、政协的监督支持下，紧紧依靠全市人民，激发争先进位的昂扬激情，凝聚跨越发展的强大合力，团结拼搏，开拓创新，务实进取，为推进惠州“尽快进入珠三角第二梯队”，为创造惠州人民更加幸福美好的生活而努力奋斗！
